# A single helicase-binding domain of DnaG couples with hexameric helicase DnaB in *Bacillus stearothermophilus*

**DOI:** 10.52601/bpr.2024.240059

**Published:** 2025-08-31

**Authors:** Hao Luo, Wenlin Liu, Yingqin Zhou, Zhongchuan Liu, Yuyang Qin, Ganggang Wang

**Affiliations:** 1 Agricultural Microbial Agents‌ Key Laboratory of Sichuan Province, Chengdu Institute of Biology, Chinese Academy of Sciences, Chengdu 610213, China; 2 University of Chinese Academy of Sciences, Beijing 100049, China

**Keywords:** DNA replication, Primosome, Helicase, Primase, Okazaki fragment

## Abstract

In bacterial DNA replication, helicase DnaB and primase DnaG form the primosome. Helicase DnaB unwinds double-stranded DNA (dsDNA) to provide templates for DNA polymerase, whereas primase DnaG supplies RNA primers to DNA polymerase for the synthesis of Okazaki fragments. How primase DnaG coordinates with helicase DnaB at the DNA replication fork remains unclear. In this study, the interactions between the helicase-binding domain of DnaG (DnaG (HBD)) and DnaB hexamer were studied. A stable ternary complex of DnaB_6_/dT_16_/DnaG(HBD) from *Bacillus stearothermophilus* was prepared and the homogeneity of the DnaB_6_/dT_16_/DnaG(HBD) complex was verified by dynamic light scattering. The stoichiometry of DnaG(HBD) to process DnaB_6_ was investigated by isothermal titration calorimetry. The results show that a single primase DnaG binds to DnaB_6_ in the presence of single-stranded DNA. Based on these results, a model is proposed to explain how the primase DnaG couples with the processing DnaB_6_ helicase during the Okazaki fragment synthesis cycle. These findings provide valuable insights into the coupling between dsDNA unwinding and RNA primer synthesis in DNA replication.

## INTRODUCTION

DNA replication in bacteria is accomplished by the replisome complex, which consists of a dozen proteins. Among them, helicase DnaB and primase DnaG form the primosome (Berger 2008; Enemark and Joshua-Tor 2006; Schlierf *et al.* 2019; Chen *et al.* 2024). DnaB unwinds the double-stranded DNA using energy provided by NTP hydrolysis, and DnaG synthesizes RNA primers for DNA polymerase to synthesize Okazaki fragments (Fernandez and Berger 2021; Kuchta and Stengel 2010). Primase DnaG consists of three domains, an N-terminal regulatory zinc-binding domain (ZBD), a central RNA polymerase domain (RPD), and a C-terminal helicase-binding domain (HBD) (Chintakayala *et al.* 2007; Chintakayala *et al.* 2008; Monachino *et al.* 2020; Rodina and Godson 2006; Syson *et al.* 2005). In *Escherichia coli*, the C-terminal octa-peptide of DnaG could bind to helicase DnaB (Tougu and Marians 1996). In *Bacillus stearothermophilus* (*B. stearothermophilus*), the primase DnaG is attached to the helicase DnaB via direct interaction with the C-terminal helicase-binding domain (HBD) (Chintakayala *et al.* 2007; Chintakayala *et al.* 2008; Syson *et al.* 2005). The interplay between DnaB and DnaG stimulates the activities of both proteins. DnaG increases both the ATPase and the helicase activities of DnaB (Kuchta and Stengel 2010), and DnaB regulates the synthesis of RNA primers by DnaG, including the synthesis efficiency and primer length (Bergsch *et al.* 2019).

The structure of the DnaB hexamer (DnaB_6_) adopts a double-layer planar ring with the NTDs in the triangular collar positioned above the ring of CTDs (Bailey *et al.* 2007; Wang *et al.* 2008). In *B. stearothermophilus*, a DnaB hexamer binds three primases, which was observed in the crystal structure of the DnaB_6_ and primase HBD complex，three primase HBD domains bind to the NTDs of DnaB hexamer (Bailey *et al.* 2007). Corn *et al*. showed that in the presence of an RNA/DNA heteroduplex, the ZBD of one primase may interact with the RPD of a second molecule in trans, which was not detected in the presence of ssDNA (Corn *et al.* 2005). In *B. stearothermophilus*, upon binding to ssDNA, DnaB_6_ forms a fibrous structure by wrapping around ssDNA to form a right-handed spiral, instead of the planar ring. The rearrangement of subunits in DnaB_6_ is hypothesized to be essential for the unwinding of dsDNA, the HBD binding sites in DnaB_6_/ssDNA complex are distinct from that in Apo DnaB hexamer (Itsathitphaisarn *et al.* 2012). Accordingly, how the primase DnaG binds to processing DnaB_6_ in each cycle of Okazaki fragment synthesis requires further investigations.

In this report, we prepared the DnaB_6_/dT_16_/DnaG(HBD) complex and provide direct evidence for the binding ratio of DnaG(HBD) to DnaB_6_ in the DnaB_6_/dT_16_/DnaG(HBD) complex. These results will advance our understanding of primosome priming on the lagging strand of *B. stearothermophilus*.

## RESULTS

### DnaG(HBD) can form a complex with DnaB_6_/dT_16_

**Figure 1:**
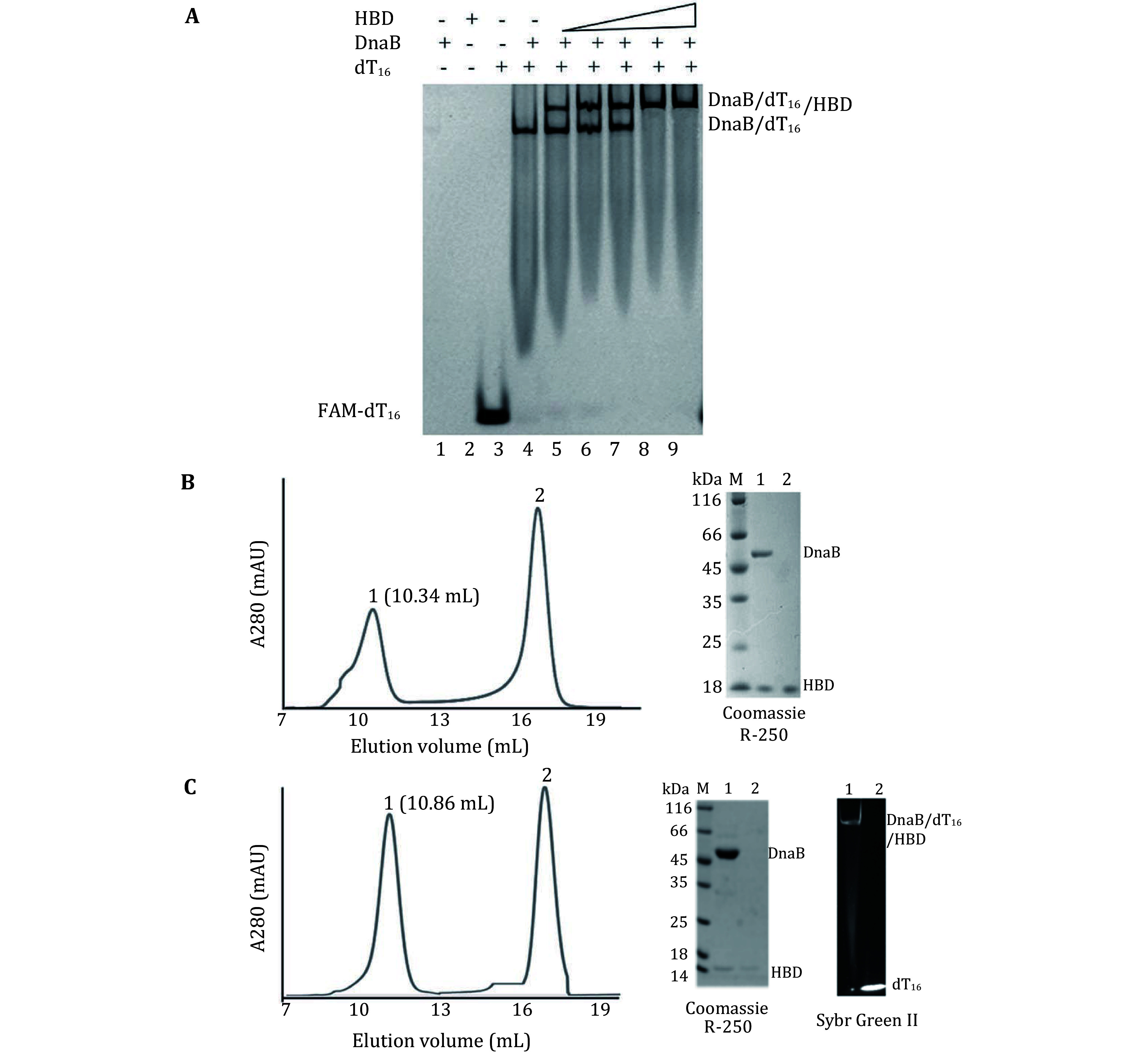
DnaG(HBD) and DnaB_6_/dT_16_ form a ternary complex. **A** EMSA experiment. Lane 1, DnaG(HBD) (10 pmol); Lane 2, DnaB (8 pmol); Lane 3, dT_16_ (10 pmol, 5'-6-FAM); Lanes 4–9, 8 pmol DnaB and 10 pmol dT_16_ were used in the binding assays and DnaG(HBD) was added at 0, 1, 3, 5, 10 and 15 pmol, respectively. Samples were analyzed using 8% native PAGE. **B** The DnaB_6_/DnaG(HBD) complex was separated by gel filtration and analyzed by SDS-PAGE. **C** The DnaB_6_/dT_16_/DnaG(HBD) complex was separated by gel filtration and analyzed by SDS-PAGE and native PAGE. The SDS-PAGE gel was stained with Coomassie R-250 and the native PAGE was stained with SYBR green. The result shows that both proteins and DNA were eluted in a single peak

The crystal structure of DnaB_6_/dT_16_ complex was previously reported, here we detected the DnaB_6_/dT_16_ binary complex through EMSA experiments (lane 4 in Fig. 1A). The addition of DnaG(HBD) resulted in the formation of a stable ternary complex (Lanes 5–9 in Fig. 1A). The DnaB_6_/HBD (Fig. 1B) and DnaB_6_/dT_16_/HBD (Fig. 1C) complexes were prepared by gel filtration chromatography, the elution volume for DnaB_6_/HBD is 10.34 mL, and 10.86 mL for DnaB_6_/dT_16_/HBD complex. 10 μL samples of the complexes were analyzed by sodium dodecyl sulfate polyacrylamide gel electrophoresis (SDS-PAGE) and Native-PAGE, and the dT_16_ in the protein/nucleic acid complex was detected by SYBR green II staining (Fig. 1C).

**Figure 2:**
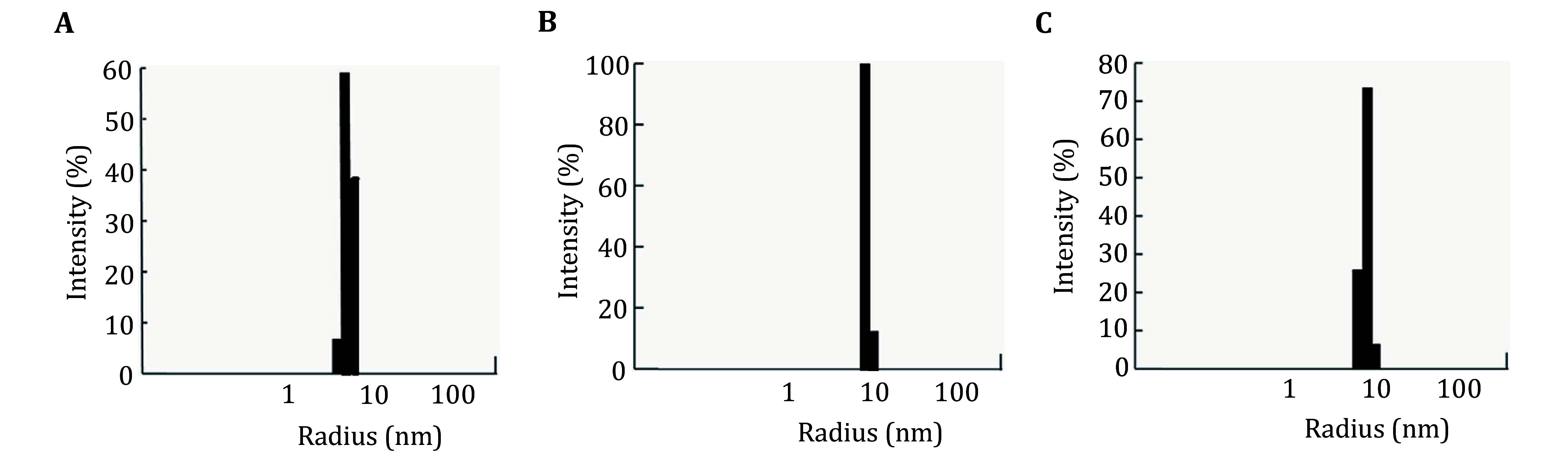
Detection of the uniformity of the complexes by dynamic light scattering. **A** DnaB_6_/DnaG(HBD). **B** DnaB_6_/dT_16_/DnaG(HBD). **C** DnaB_6_/dT_16_. The mean radii of DnaB_6_/DnaG(HBD), DnaB_6_/dT_16_/DnaG(HBD) and DnaB_6_/dT_16_ at 2 mg/mL are 6.9 ± 0.1 nm, 6.7 ± 0.1 nm and 6.6 ± 0.1 nm, respectively

The uniformity of the DnaB_6_/DnaG(HBD) and DnaB_6_/dT_16_/DnaG(HBD) complexes was verified by dynamic light scattering (DLS). Typical hydrodynamic radius distributions of the particles are shown in Fig. 2. Particle sizes were obtained from cumulant analysis of the measured DLS correlation functions and thus represent an average over the whole size distribution. Average hydrodynamic radii (from cumulant analysis) of DnaB_6_/DnaG(HBD) and DnaB_6_/dT_16_/DnaG(HBD) were determined to be 6.9 ± 0.1 nm and 6.7 ± 0.1 nm, respectively (Fig. 2).

### The proportion of helicase to primase in DnaB_6_/dT_16_/HBD complex

**Figure 3:**
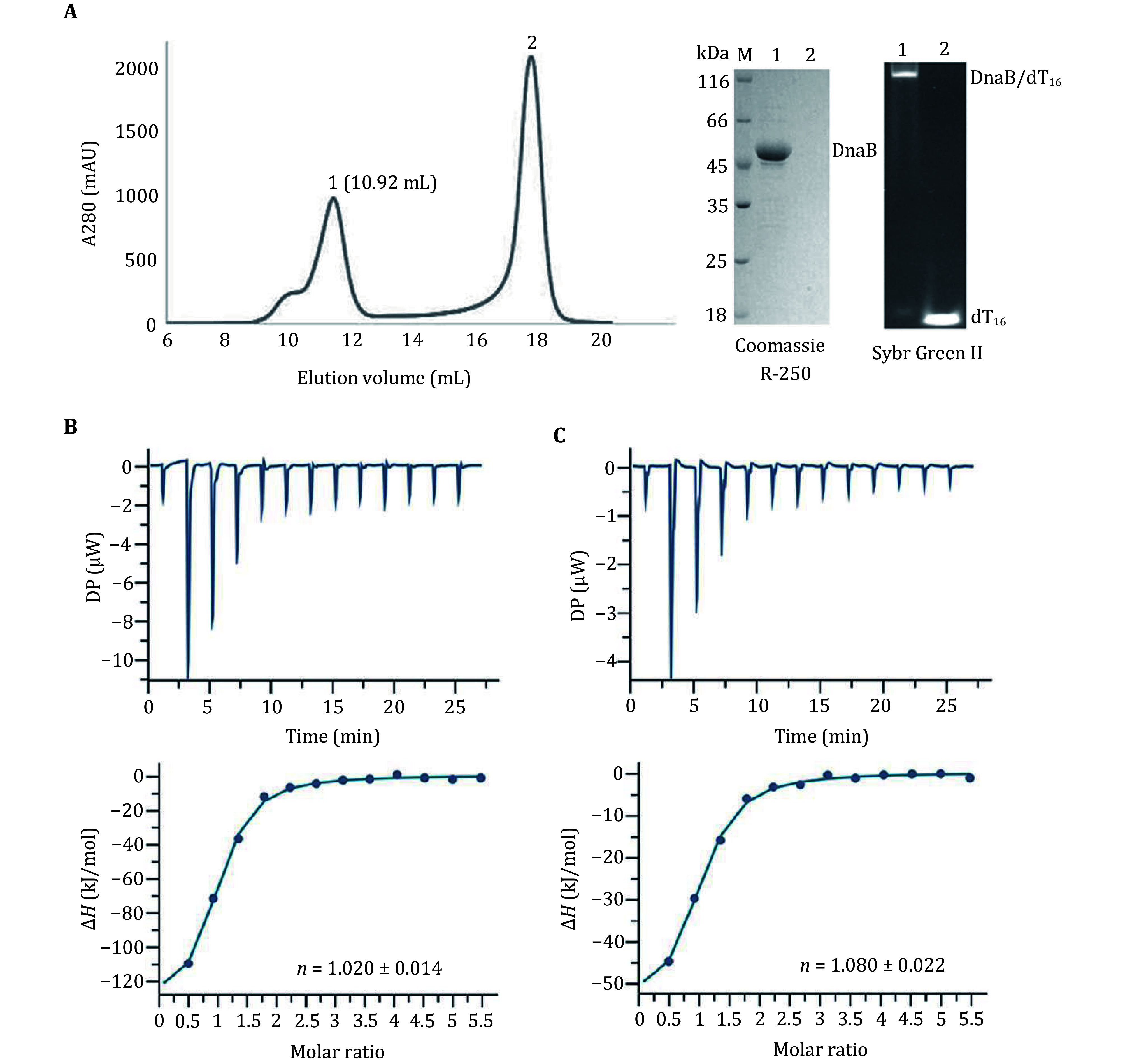
ITC measurements of the interactions between DnaG(HBD) and DnaB_6_/dT_16_ complex. **A** Preparation and detection of the DnaB_6_/dT_16_ complex. The DnaB_6_/dT_16_ complex was separated by gel filtration and analyzed by SDS-PAGE. The gel was stained initially with Coomassie R-250 and then with SYBR green. The results show that the DnaB_6_/dT_16_ binary complex was prepared successfully. **B** The upper panel represents a typical raw trace of the titration of DnaG(HBD) to the DnaB_6_/dT_16_ complex at 25°C. The lower panel shows the result of an integration of heat exchange for each injection fitted with the one-set-of-sites model using the MicroCal PEAQ-ITC analysis software supplied by the manufacturer. **C** Reverse titration of the DnaB_6_/dT_16_ complex to DnaG(HBD). The upper panel represents a typical calorimetric titration and the lower panel shows the resulting integrated binding isotherm at 25°C

DnaB_6_ has been proposed to adopt continuous subunit rearrangements during translocation (Itsathitphaisarn *et al.* 2012). This raises the question as to how primase DnaG coordinates with the dynamic DnaB_6_ for priming because DnaG binding sites may be formed and used sequentially. To resolve this question, Isothermal titration calorimetry (ITC) was used to study the thermodynamic aspects of the binding between DnaG(HBD) and the DnaB_6_/dT_16_ complex. The DnaB_6_/dT_16_ complex was prepared by gel filtration chromatography. The binary complex was analyzed by SDS-PAGE gel and the dT_16_ in the protein/nucleic acid complex was detected by SYBR green II staining (Fig. 3A). The homogeneity of the DnaB_6_/dT_16_ complex was verified by DLS (Fig. 2C). The ITC measurements were performed to evaluate the binding ratio of DnaG(HBD) to DnaB_6_/dT_16_ complex. The binding isotherm and plotted titration curve for the binding of DnaG(HBD) to DnaB_6_/dT_16_ is shown in Fig. 3B. The data fit best to a single binding site model, yielding a binding stoichiometry of one DnaG(HBD) per DnaB_6_/dT_16_ complex. Similar results were obtained in the reverse titration (Fig. 3C). At the same time, the DnaB hexamer was titrated by DnaG(HBD), and the data results in the binding ratio of three DnaG(HBD) per DnaB hexamer in the absence of ssDNA. The stoichiometry(*n*), equilibrium dissociation constant (*K*_d_) and the thermodynamic parameters obtained from at least three independent ITC experiments are summarized in Table 1.

**Table 1:** Thermodynamic parameters for the interaction between DnaB_6_/dT_16_ and DnaG(HBD)

Complexes	*n*	*K*_d_ (μmol/L)	Δ*H* (kJ/mol)	Δ*G* (kJ/mol)	TΔ*S* (kJ/mol)
HBD-DnaB_6_/dT_16_(1)	1.020 ± 0.014	1.13 ± 0.22	–94.9 ± 2.21	–35.5	64.5
HBD-DnaB_6_/dT_16_(2)	0.981 ± 0.026	1.07 ± 0.13	–93.0 ± 4.39	–34.7	58.3
HBD-DnaB_6_/dT_16_(3)	1.090 ± 0.073	0.96 ± 0.26	–95.3 ± 6.9	–30.6	64.7
DnaB_6_/dT_16_-HBD(1)	0.987 ± 0.049	1.01 ± 0.33	–46.7 ± 3.99	–33.4	13.3
DnaB_6_/dT_16_-HBD(2)	1.080 ± 0.022	1.11 ± 0.24	–38.4 ± 1.28	–35.0	3.37
DnaB_6_/dT_16_-HBD(3)	1.050 ± 0.022	0.89 ± 0.16	–40.8 ± 5.32	–31.5	9.3

In bacteriophage T7, the primase is linked covalently to the helicase as a bifunctional protein. Thus, six primase domains stack onto the helicase ring at the DNA replication fork (Gao *et al.* 2019; Kulczyk *et al.* 2017). In *B. stearothermophilus*, three DnaG (HBD) molecules bind to the NTD trimer interface of the DnaB_6_ ring (Bailey *et al.* 2007). When DnaB binds to ssDNA, a helical filament shape instead of a ring was observed (Itsathitphaisarn *et al.* 2012), and the conformational change led to the disruption of the DnaG (HBD) binding sites. Accordingly, less of DnaG (HBD) binds to the DnaB/dT_16_ complex.

## DISCUSSION

In this report, ITC analysis was used to show that one primase interacts with the helicase in the presence of ssDNA. We believe that this is the first time the stoichiometry of primase DnaG to DnaB_6_ in the DnaB_6_/dT_16_/DnaG(HBD) complex has been validated experimentally. In eukaryotic DNA replication, one primase was also observed to associate with the DNA polymerase for lagging strand synthesis (Sun *et al.* 2015). In *E. coli*, priming is initiated once per 1–3 kb of genomic DNA. DnaG is recruited to the replication fork via HBD binding to DnaB_6_, which recognizes a specific initiation site to produce an RNA primer (Bergsch *et al.* 2019; Chen *et al.* 2024; Jameson and Wilkinson 2017; Wu *et al.* 1992). After priming, the primase subsequently interacts with the clamp loader for the primer hand-off to DNA polymerase and then leaves the fork (Chang and Marians 2000; Tougu *et al.* 1994; Wu *et al.* 1992). Therefore, the 1:1 stoichiometry of DnaG(HBD) to DnaB_6_ coordinates the distributive action of DnaG with the structural rearrangement of processing DnaB_6_ in the cycle of Okazaki fragment synthesis.

**Figure 4:**
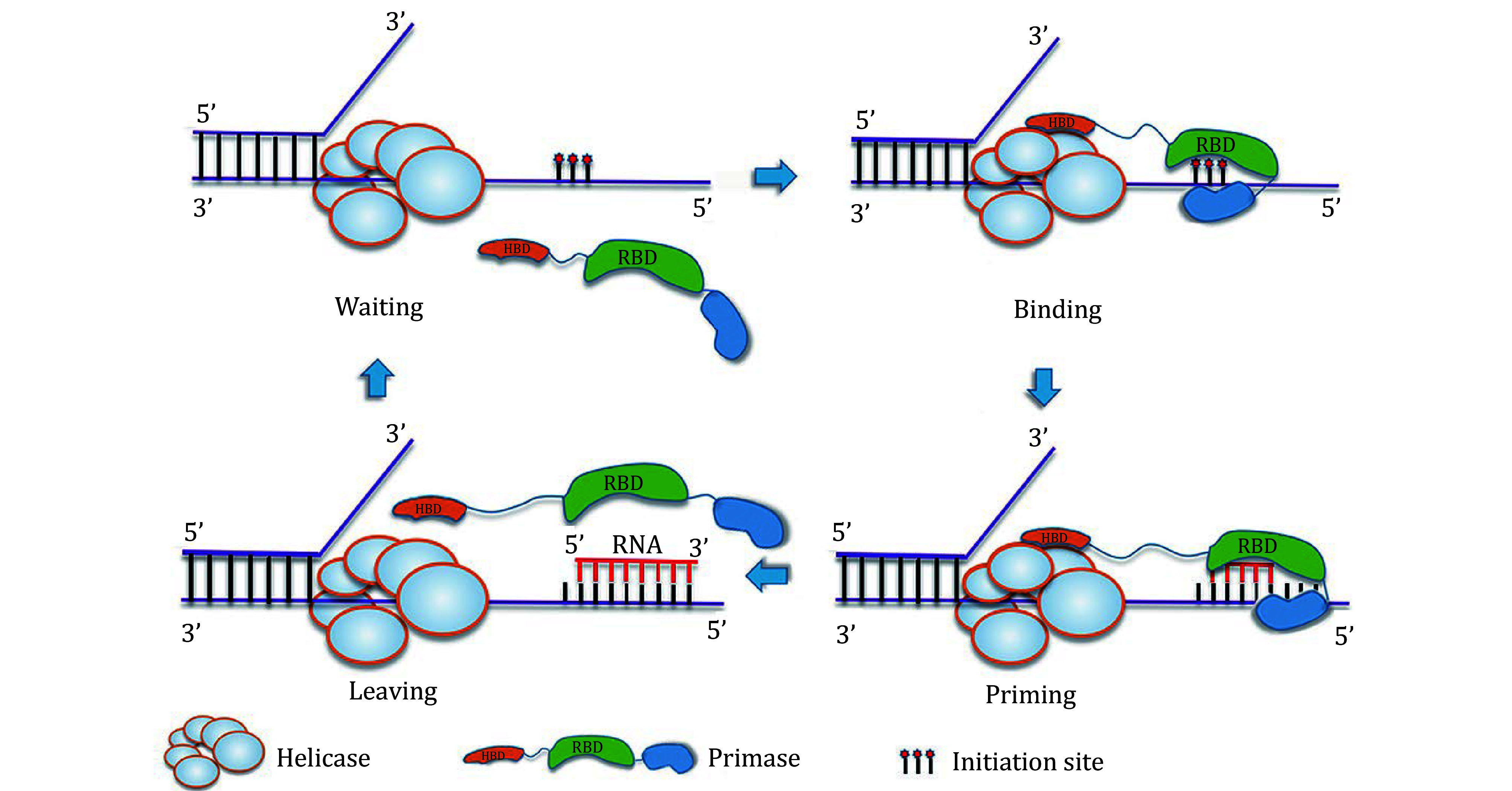
A model of the primosome priming on the lagging strand in *B. stearothermophilus*

By combining our data with the previous biochemical and structural results (Chang and Marians 2000; Itsathitphaisarn *et al.* 2012; Tougu *et al.* 1994; Luo, *et al.* 2019), a model of the primase DnaG at the DNA replication fork in *B. stearothermophilus* can be created. We propose that in the presence of a priming signal one primase DnaG is recruited to the DNA replication fork by HBD binding to the DnaB_6_ helicase. Subsequently, the ZBD/RPD recognizes a specific initiation site and moves away for priming and the RNA primer is passed to the DNA polymerase (Lee *et al.* 2012). After priming, the HBD binding site on DnaB_6_ undergoes a conformational change that causes primase DnaG to disassociate from DnaB_6_ (Itsathitphaisarn *et al.* 2012) (Fig. 4). In this mode of primosome organization, the distance between HBD and the ZBD/RPD may increase during primer elongation. The 30-residue linker between HBD and ZBD/RPD should facilitate structural reorganization of the ZBD/RPD to accomplish primer synthesis (Luo *et al.* 2019). Although this model is based on data from *B. stearothermophilus*, it may be a representative of other pathogen bacteria, such as *E. coli* and *Staphylococcus aureus*, since the counterparts of helicase DnaB and primase DnaG are conserved in these bacteria (Chang and Marians 2000; Kuchta and Stengel 2010; Patel *et al.* 2011; Wang *et al.* 2008). Conversely, bacterial helicase DnaB and primase DnaG are quite distinct from their counterparts found in humans (Baranovskiy *et al.* 2015; Baranovskiy *et al.* 2016). Thus, the primosome represents an ideal target for the development of novel antibiotics (Ilic *et al.* 2018). The data reported here should facilitate the design of new classes of antibiotics that target the DnaG binding site.

In conclusion, the results presented define the architecture of the primosome in *B. stearothermophilus* and provide insights into how the helicase and primase in DNA replication are physically and functionally coupled. This work also paved the curb for exploring the helicase and primase interplay *in vivo*. To better define the chemical basis for helicase and primase coupling, further investigations are required to determine the three-dimensional structure of DnaB_6_/ssDNA/DnaG ternary complexes.

## MATERIALS AND METHODS

### Protein expression and purification

The DnaB and DnaG(HBD) proteins were prepared as described in the previous publications (Pan *et al.* 1999; Yang and Wang 2016; Zhou *et al.* 2017). Briefly, the PCR amplified genes dnaB, and dnaG (hbd) from *B. stearothermophilus* were inserted into the pGEX-6p-1 vector. The primers of DnaB-F (5΄-CGCGGATCCTATGAGCGAGCTGTTTTCAGAA-3΄), HBD-F (5΄-CGCGGATCCAAGTTGCTGCCGGCTTTTCA- 3΄) with BamHI site and DnaB-R (5΄-CCGCGCCTCGAGTTACGCCCCCGGCGGAATTTG-3΄), HBD-R (5΄-CCGCGCCTCGAGTTATGAGGAAGATAACATTT-3΄) with XhoI site were made (Sangon Biotech Co., Ltd). All constructs were confirmed by DNA sequencing. The positive constructs were transformed into *E. coli* BL21 (DE3) cells for expression of the recombinant proteins. The cells were grown to an OD_600_ of 0.4–0.6 in LB medium containing 100 μg/mL ampicillin at 37°C. Overexpression of these proteins was induced by the addition of IPTG to a final concentration of 0.2 mmol/L, and cells were grown for a further 16 h at 16°C. For purification of recombinant proteins, *E. coli* cells were harvested by centrifugation, and the cells were resuspended in Buffer A (25 mmol/L Tris-HCl, pH 8.0, 300 mmol/L NaCl, 1 mmol/L DTT) and lysed by sonication. After clarification by centrifugation, the GST-fusion proteins were isolated by Sepharose 4B affinity chromatography, and the GST tag was removed by digestion with PreScission protease at 4°C overnight. Proteins were then purified using source 15Q (GE, USA) ion-exchange chromatography followed by passage through a Superdex-200/Superdex-75 gel-filtration column. The proteins were concentrated to ~10 mg/mL in Buffer B (25 mmol/L Tris-HCl, pH 8.0, 100 mmol/L NaCl, 1 mmol/L DTT) for further experiments.

### Electrophoretic mobility gel shift assay (EMSA)

The formation of DnaB/dT_16_/DnaG(HBD) complex was resolved by EMSA experiments. The 8 pmol DnaB was mixed with 25% molar excess dT_16_ and kept at 4°C for 30 min, and then the DnaG(HBD) was added for incubation at 4°C for 20 min, the DnaG(HBD) was added at 1, 3, 5, 10 and 15 pmol, respectively. The binding buffer contained 25 mmol/L Tris pH 8.0, 100 mmol/L NaCl, 10% glycerol, 1 mmol/L DTT, 5 mmol/L MgCl_2_ and 2 mmol/L ATP, the total reaction volume was 20 µL. The samples were analyzed using 6% Native-PAGE. The gel was photographed using a Geldoc XR+ system (Bio-rad, Hercules, CA). The DNAs used in EMSA were single-stranded DNA labeled at the 5'-end with 6-carboxyfluorescein (6-FAM) (Sangon Biotech Co., Ltd).

### Preparation of the complexes

30 μmol/L DnaB_6_ (100 μL) in Buffer C (25 mmol/L Tris-HCl, pH 8.0, 100 mmol/L NaCl, 1 mmol/L DTT, 5 mmol/L MgCl_2_) was incubated with 5-fold molar excess of HBD (25 μL) for 30 min at 4°C to form the complex. The complex of DnaB_6_/dT_16_ was prepared by incubating 30 μmol/L of DnaB_6_ (100 μL) in Buffer C with 1 mmol/L ATPγS and 3-fold molar excess of ssDNA for 30 min at 4°C. As to the complex of DnaB_6_/dT_16_/DnaG(HBD), 30 μmol/L of DnaB_6_ (100 μL) in Buffer C was incubated with 1 mmol/L ATPγS and 3-fold molar excess of ssDNA for 30 min at 4°C. Subsequently, 5-fold molar excess of HBD (25 μL) was added and this solution was incubated for 30 min at 4°C. Samples were replenished to 150 μL with Buffer C and purified by Superdex™ 200 10/300 GL size exclusion chromatography (GE, USA) (Bailey *et al.* 2007; Itsathitphaisarn *et al.* 2012). The concentration of the complex samples was determined by a Nanodrop lite UV-Vis spectrophotometer (Thermol, USA) (Chen *et al.* 2021). 10 μL samples of complexes purified by Superdex™ 200 10/300 GL were detected by denaturing and non-denaturing gel electrophoresis, and the nucleic acids in the complexes, as well as displaced nucleic acids were colored by SYBR green II. And then the 500 μL samples in the middle of the peaks were concentrated for ITC analysis.

### Dynamic light scattering (DLS) measurements

The size of the samples was determined by DLS measurements in a 50-μL low volume quartz cuvette using a Dynapro Nanostar (Wyatt, Beijing, China) at 25°C. The distribution represents the average of five measurements, and each measurement consisted of ten 0.5-s acquisitions. The time-dependent correlation function *G*(τ) was measured for different samples in Buffer C. *D*_t_ is related to the hydrodynamic radius *R*_h_ of particles through the Stokes-Einstein relation *D*_0_ = *k*_B_*T*/6π*ηR*_h_, where *k*_B_ is Boltzmann’s constant (1.381 × 10^–23^ J/K), *T* is the absolute temperature and *η* is the absolute (or dynamic) viscosity of the solvent (Bordi *et al.* 2001; Chiasserini *et al.* 2015; Dahani *et al.* 2015). The measured autocorrelation functions were analyzed using the cumulants method to evaluate the uniformity and average sizes of the particles (Frisken 2001).

### Isothermal titration calorimetry (ITC) measurements

ITC was employed to measure the stoichiometric ratio and binding affinities of two components. All samples were prepared in a buffer containing 25 mmol/L Tris, pH 8.0, 100 mmol/L NaCl. The samples were centrifuged to remove any precipitate before the experiments. All measurements were carried out at 25°C by using a PEAQ-ITC instrument (Malvern, UK). The sample concentrations in the titration needle and sample cell were 200 μmol/L and 10 μmol/L, respectively. Each set of titration experiments was repeated three times. The binding curves were analyzed, and dissociation constants (*K*_d_) were determined by a “one-set-of-sites” model using the MicroCal PEAQ-ITC analysis software supplied by the manufacturer (Malvern, UK).

## Conflict of interest

Hao Luo, Wenlin Liu, Yingqin Zhou, Zhongchuan Liu, Yuyang Qin and Ganggang Wang declare that they have no conflict of interest.
